# Variation in *OPA1* does not explain the incomplete penetrance of Leber hereditary optic neuropathy

**Published:** 2010-12-15

**Authors:** Gavin Hudson, Patrick Yu-Wai-Man, Phillip G. Griffiths, Leonardo Caporali, Solange S. Salomao, Adriana Berezovsky, Valerio Carelli, Massimo Zeviani, Patrick F. Chinnery

**Affiliations:** 1 Institute for Human Genetics, Newcastle University, Newcastle upon Tyne, UK; 2 Department of Ophthalmology, Royal Victoria Infirmary, Newcastle upon Tyne, UK; 3 Department of Neurological Sciences, University of Bologna, Bologna, Italy; 4 Departamento de Oftalmologia, Universidade Federal de Sao Paulo, UNIFESP, Sao Paulo, Brazil; 5 Foundation Neurological Institue C. Besta, Unit of Molecular Neurogenetics, Milan, Italy

## Abstract

**Purpose:**

Leber hereditary optic neuropathy (LHON) is a common cause of inherited blindness, primarily due to one of three mitochondrial DNA (mtDNA) mutations. These mtDNA pathogenic mutations have variable clinical penetrance. Recent linkage evidence raised the possibility that the nuclear gene optic atrophy 1 (*OPA1*) determines whether mtDNA mutation carriers develop blindness. To validate these findings we studied *OPA1* in three independent LHON cohorts: sequencing the gene in discordant male sib pairs, carrying out a family-based association study of common functional genetic variants, and carrying out a population-based association study of the same genetic variants.

**Methods:**

We tested 3 hypothesis in three separate study groups. Study group 1: Direct sequencing of *OPA1* coding regions was performed using sequencing methodologies (Applied Biosystems, Foster City, CA). Chromatograms were compared with the GenBank reference sequence NM_015560.1. Splice-site prediction was performed using GeneSplicer. Study group 2: Genotyping for rs166850 and rs10451941 was performed by restriction fragment length polymorphism (RFLP) analysis with specific primers for both genotypes, using The restriction enzymes RsaI and FspBI to discriminate genotypes. Study group 3: Genotyping for rs166850 and rs10451941 was performed by primer extension of allele-specific extensions products by matrix-associated laser desorption/ionisation time-of-flight (MALDI-TOF, Seqeunom, San Diego, CA) mass spectrometry. Allele and genotype frequencies were compared using Pearson’s chi-square test. Multiple logistic regression was performed to look for interactions between the variables. All analyses were performed using SPSS software version 17.0 (SPSS Inc.).

**Results:**

In all three groups we were unable to find an association between *OPA1* genetic variation and visual failure in LHON mtDNA mutation carriers.

**Conclusions:**

Our findings suggest that genetic variation in *OPA1* is unlikely to make a major contribution to the risk of blindness in LHON mutation carriers.

## Introduction

Leber hereditary optic neuropathy (LHON, OMIM 535000) is a common cause of inherited blindness that typically presents with bilateral, painless, subacute visual failure in young adult males [1]. Affected individuals develop focal degeneration of the optic nerve and present clinically with impaired color vision (dyschromatopsia), a dense visual field defect (central or cecocentral scotoma), and abnormal visual electrophysiology due to primary retinal ganglion cell loss [1]. The diagnosis is usually confirmed by molecular genetic analysis for one of three common mitochondrial DNA (mtDNA) mutations, which all affect genes coding for complex I subunits of the respiratory chain: m.3460G>A, m.11778G>A, and m14484T>C. However, not all patients harboring a pathogenic LHON mtDNA mutation develop visual failure [2]. Segregation analysis of LHON pedigrees implicates a two-locus model, with the mtDNA mutation as one locus, together with a modulating chromosomal locus [3]. Attempts to identify a nuclear modifying gene by both genetic mapping and functional genomics have been inconclusive [4-6]. Recently, a region of chromosome 3 was identified as important in determining the risk of visual loss in LHON [7]. Although this work led to a report associating the presenilin associated, rhomboid-like (*PARL*) gene with the LHON phenotype, the same linkage region harbors optic atrophy 1 (*OPA1*), a gene critical to mitochondrial function. This raises the possibility that the *PARL* association was mediated through linkage disequilibrium with the *OPA1* gene.

*OPA1* is a nuclear gene coding for a mitochondrial protein critical for mtDNA maintenance, effective oxidative phosphorylation, and maintenance of the mitochondrial network [8-10]. Mutations in *OPA1* are a principle cause of autosomal dominant optic atrophy (DOA) [11-13]. Like LHON, DOA has a markedly variable clinical phenotype, and both disorders share the selective loss of retinal ganglion cells [14]. Common genetic variants in *OPA1,* including rs166850 and rs10451941, have been associated with normal tension glaucoma [15,16], a disorder which also shares the selective loss of retinal ganglion cells with LHON and DOA [16]. *OPA1* expression also appears to be downregulated in LHON patients [17].

With this in mind, we tested the following two hypotheses in three independent cohorts. (1) Cosegregating pathogenic mutations in *OPA1* might explain why only approximately 40% of men harboring pathogenic LHON mtDNA mutations develop visual failure. This was achieved by sequencing the entire coding region of *OPA1* in pairs of clinically discordant male siblings. (2) Two functional *OPA1* SNPs, rs166850 and rs10451941, which were previously implicated in the pathogenesis of optic neuropathy [16], might modulate the phenotype in LHON pedigrees. This was achieved by carrying out: (a) a family-based association study of a large, well-characterized LHON pedigree, and (b) a population-based association study of LHON mutation carriers from across Europe. Given the established role of mtDNA haplogroups in modulating the clinical presentation of specific LHON mutations [18], our analysis incorporated previously determined haplogroup data.

## Methods

### Subjects

Hypothesis 1 was tested in 16 discordant sib pairs. Hypothesis 2 was tested in a large Brazilian m.11778G>A LHON pedigree [19] (study group 2; clinically affected, n=23; clinically unaffected, n=39) as well as in an independent cohort of 248 LHON mtDNA mutation carriers (study group 3: clinically affected, n=95; clinically unaffected, n=153) from centers around Europe. The clinical phenotype was determined by a local ophthalmologist, and all subjects were homoplasmic for one of the three primary LHON mtDNA mutations (study group 1 - clinically affected mt.11778G>A=16 and clinically unaffected mt.11778G>A=16; study group 2 - clinically affected mt.11778G>A=23 and clinically unaffected mt.11778G>A=39; study group 3 - clinically affected, mt.3460G>A=12, mt.11778G>A=81, mt.14484T>C=2 and clinically unaffected, mt.3460G>A=26, mt.11778G>A=126, mt.14484 T>C=1). This was confirmed by direct sequencing of the relevant mitochondrial NADH dehydrogenase genes (*MTND*-1, −4 and −6) or by PCR-restriction fragment-length polymorphism analysis, as previously described [20]. Unaffected carriers were classified and included only if they had remained asymptomatic until over 30 years of age.

### Molecular studies

#### Study group 1

Direct sequencing of *OPA1* coding regions was performed using sequencing methodologies (Applied Biosystems, Foster City, CA). Sequence chromatograms were directly compared with the appropriate GenBank reference sequence (NM_015560.1) using SeqScape software (v2.1, Applied Biosystems). Splice-site prediction was performed using GeneSplicer [21].

#### Study group 2

Genotyping for rs166850 and rs10451941 was performed by restriction fragment-length polymorphism analysis with specific primers for both genotypes (sense, 5′-CCC TTT TAG TTT TTA CGA TGA AGA-3′; antisense, 5′-TTG CTT AAG ACA TTA CTT GGA ACA-3′). The restriction enzymes RsaI and FspBI (Fermentas, York, UK) discriminated the respective genotypes.

#### Study group 3

Genotyping for rs166850 and rs10451941 was performed by primer extension of allele-specific extension products by matrix-associated laser desorption/ionization time-of-flight mass spectrometry (MALDI-TOF; Seqeunom, San Diego, CA). Genotypes were confirmed in 10% of random samples, by direct sequencing using a genetic analyzer (ABI3100; Applied Biosystems).

### Statistical analysis

Allele and genotype frequencies were compared using Pearson’s chi-square test. Multiple logistic regression was performed to look for interactions between the variables. All analyses were performed using the Statistical Package for Social Sciences software (version 17.0; SPSS Inc., Middlesex, UK).

## Results

### *OPA1* sequencing in study group 1

Sequencing of *OPA1* coding regions in LHON sib pairs did not identify any previously reported pathogenic mutations in LHON sib pairs [22]. Seven previously reported synonymous polymorphisms were identified (rs7624750, rs60201300, rs34307082, rs10451941, rs9831900, rs9851685, and rs10937595) [23]; however, none segregated with the disease either individually (Table 1) or in different combinations, and no variant was predicted to disrupt exon splicing. The frequency of these variants was not significantly different from that of the background population.

**Table 1:** Allelic frequency comparison of SNPs identified in study group 1, discordant LHON sib-pairs (where A=affected, U=unaffected sibling and P is uncorrected probability by Fishers Exact test).

**SNP**	**A (n=32)**	**U (n=32)**	**p**
rs7624750	7	5	0.750
rs60201300	21	19	0.079
rs34307082	8	8	1.000
rs10451941	21	19	0.079
rs9831900	21	19	0.079
rs9851685	7	5	0.750
rs10937595	21	19	0.079

The table shows no significant association to *OPA1* variants and disease.

### rs166850 and rs10451941 genotyping

#### Study group 2

There was no association between either rs166850 or rs10451941 and visual failure in the large Brazilian m.11778G>A pedigree for both alleles, independently (Pearson’s p=0.669 and p=1.00, respectively; Table 2) or for the genotypes (Pearson’s p=0.491 and p=0.966, respectively; Table 2).

**Table 2:** Analysis of *OPA1*: rs166850 and rs10451941 frequencies in study group 2, the Brazilian LHON pedigree (where, A=affected; U=unaffected, and P in uncorrected probability by Pearson’s chi-square test).

**SNP**	**Status**	**C**	**T**	**p**	**CC**	**CT**	**TT**	**p**
rs166850	A	43	3	0.669	20	3	0	0.491
	U	75	3		36	3	0	
rs10451941	A	25	21	1.000	8	9	6	0.966
	U	42	36		14	14	11	

The table shows no significant association to either OPA1 alleles or genotypes in study group 2.

#### Study group 3

There was no association between rs166850 or rs10451941 and visual failure in the European LHON mutation carriers when comparing alleles (Pearson’s p=0.115 and p=1.00, respectively; Table 3) or genotypes (Pearson’s p=0.161 and p=0.959, respectively; Table 3). As previous studies have shown, the rs166850: rs10451941 CT:TT genotype is associated with increased risk of developing normal tension glaucoma (NTG), which, comparable to LHON, preferentially affects the retinal ganglion cell [16]. We therefore performed analyses of rs166850: rs10451941 complex genotypes versus LHON (Table 4). Tests of association did not reveal any link between any complex genotype and visual loss in subjects harboring a pathogenic LHON mutation.

**Table 3:** Analysis of *OPA1*: rs166850 and rs10451941 frequencies in study group 3, European LHON mutation carriers (where, A=affected; U=unaffected, and P in uncorrected probability by Pearson’s chi-square test).

**SNP**	**Status**	**C**	**T**	**p**	**CC**	**CT**	**TT**	**p**
rs166850	A	156	34	0.115	64	28	3	0.161
	U	268	38		116	36	1	
rs10451941	A	99	91	1.000	24	51	20	0.959
	U	159	147		37	85	31	

The table shows no significant association to common OPA1 variants and disease in study group 3.

**Table 4:** Further analysis of *OPA1* rs166850:rs10451941complex genotype frequencies in study group 3, European LHON mutation carriers (where, A=affected; U=unaffected and P is uncorrected probability by Fishers Exact test).

rs166850 **:rs10451941**	**A**	**U**	**p**
CC:CC	22	37	0.8794
CC:CT	28	56	0.2717
CC:TT	14	23	1.0000
CT:CC	1	0	0.3831
CT:CT	23	30	0.4272
CT:TT	4	6	1.0000
TT:CC	1	0	0.3831
TT:CT	1	0	0.3831
TT:TT	1	1	1.0000

Analysis of complex genotypes found no significant association to disease in study group 3.

### Relationship to mitochondrial DNA haplogroup J

The clinical penetrance of the LHON mutations m.11778G>A and m.14484T>C is strongly associated with mitochondrial haplogroup J [18]. With this is mind, we compared *OPA1* rs166850 and rs10451941 genotypes in both LHON J and non-J haplogroups by logistic regression analysis, which controls for age, gender, and the specific LHON mtDNA mutation. This failed to identify an interacting association between the background mitochondrial haplogroup J, the *OPA1* rs166850 and rs10451941 genotypes, and the incidence of visual failure in LHON patients (p values: rs166850 genotype, p=0.464; for rs10451941 genotype, p=0.801; for combined complex genotypes, p=0.616). There was also no significant association identified in non-J haplogroups.

## Discussion

This study was designed to test the hypothesis that genetic variation in *OPA1* is the major factor determining why only the minority of LHON mtDNA mutation carriers develop visual failure. Our study of three independent cohorts found no evidence to support this hypothesis. Whole-gene sequencing in discordant sib pairs did not identify a pathogenic *OPA1* variant. The polymorphic variants that were identified were synonymous and poorly conserved in the population (mean population heterozygosity; rs35801538=0.031, rs9851685=0.495, and rs35540805=0.027, dbSNP, [23]), and not previously associated with visual failure. Unlike glaucoma, we were unable to identify an association between the rs166850: rs10451941 genotype and the risk of visual impairment in a large, well characterized Brazilian LHON pedigree (study group 2). Being maternally related, all of the Brazilian mutation carriers harbored the same LHON mtDNA mutation (m.11778G>A) and belonged to the same mtDNA background haplogroup (J) [19]. Given the well established relationship between visual loss and both the specific mtDNA mutation and the background mtDNA haplogroup [18], we studied a third group and determined the relationship between the clinical phenotype and both rs166850 and rs10451941 in isolation; a multivariate analysis accounted for the specific primary mtDNA LHON mutation and the mtDNA haplogroup (study group 3). Once again we found no evidence of an association between common functional genetic variants of *OPA1* and visual failure in LHON mtDNA mutation carriers.

Having tested the hypothesis in several different ways and in different cohorts, we conclude that it is unlikely that genetic variation in *OPA1* makes a major contribution to the risk of blindness in LHON mutation carriers. Although we cannot exclude a more subtle genetic effect, such as an influence on the age at onset of the visual failure or chance of recovery, either directly or through an interaction with other genes, a much larger study is required to address this issue. This may not be technically possible, given the relative rarity of LHON mtDNA mutation carriers in the general population. Based on the results presented here, it seems more likely that other genetic and environmental factors explain the variable penetrance of this mtDNA disorder [24].
